# EphB2 activation is required for ependymoma development as well as inhibits differentiation and promotes proliferation of the transformed cell

**DOI:** 10.1038/srep09248

**Published:** 2015-03-24

**Authors:** Phylip Chen, Nathan Rossi, Samuel Priddy, Christopher R. Pierson, Adam W. Studebaker, Robert A. Johnson

**Affiliations:** 1 Center For Childhood Cancer and Blood Diseases, The Research Institute at Nationwide Children's Hospital; 2 Department of Pathology and Laboratory Medicine, Nationwide Children's Hospital and Department of Pathology, The Ohio State University College of Medicine; 3 Department of Pediatrics, The Ohio State University College of Medicine

## Abstract

Our intracranial implantation mouse model of ependymoma clearly demonstrates overexpression of the ephrin receptor EphB2 in *Ink4a/Arf^(−/−)^* supratentorial embryonic neural stem cells (STeNSCs) to be essential for transformation and disease development; however the requirement for and consequence of receptor activation on transformation and neural stem cell function were not examined. We definitively illustrate the necessity for receptor activation in cellular transformation and the importance of implantation site and microenvironment in directing ependymoma development. *In vitro* assays of EphB2 overexpressing *Ink4a/Arf^(−/−)^* STeNSCs showed no changes in their neural stem cell characteristics (stem cell marker expression and self-renewal) upon receptor activation, but EphB2 driven tumor cells were inhibited significantly in differentiation and exhibited increased tumorsphere formation and cellular proliferation in response to ephrin-B ligand mediated receptor activation. Additionally, we observed substantial differences in the phosphorylation state of several key proteins involved in Ras and p38 MAPK signaling when comparing EphB2 overexpressing *Ink4a/Arf^(−/−)^* STeNSCs and tumor cells with relatively little change in total protein levels. We propose that EphB2 mediated ependymoma development is a multifactorial process requiring microenvironment directed receptor activation, resulting in changes in the phosphorylation status of key regulatory proteins, maintenance of a stem-like state and cellular proliferation.

EphB2 is a member of the membrane bound Ephrin (Eph) family of receptor tyrosine kinases (RTK) consisting of 16 members divided into two groups: EphA (EphA1-10) and EphB (EphB1-6). The receptor is activated when it interacts with its cognate membrane-bound ligand (ephrins A1–6 or ephrins B1–3) on an opposing cell causing receptor/ligand dimerization, receptor kinase domain autophosphorylation and interactions with Src homology 2 domain (SH2 domain) containing signaling proteins that initiate downstream pathways via forward signaling^1^,^2^,^3^,^4^. While less studied, activation-induced phosphorylation of the receptor's C-terminal PDZ/SAM domain is involved in self-association and protein-protein interactions contributing to its function^5^,^6^. Additionally, ephrin-B ligands undergo a process known as reverse signaling whereby receptor interaction induces phosphorylation of the ligands intracellular PDZ domain allowing for interactions with SH2 and PDZ containing proteins and signal transmission in the ligand expressing cell. Eph signaling triggers multiple pathways, most notably the Ras (R-Ras, H-Ras, N-Ras) and Rho (Rho, Rac1, Cdc42) family of small GTPases, making it critical to many cellular functions including cellular proliferation, cytoskeletal dynamics and cell adhesion^2^,^3^,^4^.

Aberrant Eph expression (increases or decreases) is observed in many human cancers in particular colorectal, prostate, breast cancers and interestingly in melanomas and gliomas where it correlates with patient survival, tumor prognosis, grade, metastasis, migration and invasion^3^,^7^,^8^,^9^,^10^,^11^,^12^. Several studies have demonstrated a RhoA mediated increase in melanoma cell migration when either EphA2 or EphA4 are overexpressed^13^,^14^,^15^,^16^. Escalation in glioma tumor cell migration, growth and adhesion have been attributed to elevated EphB2 levels and R-Ras activation^17^,^18^,^19^,^20^. Additionally, loss of EphB2 expression has been implicated in the progression of colorectal cancer^21^. As these studies illustrate, the current understanding of the connection between Eph activity and cancer is based predominantly on observing the impact that modulating receptor/ligand interaction or expression levels has on the survival, migration or proliferation of an established tumor. Although very informative, these studies were not designed to examine the potential function ephrin signaling has in tumor development. We have created a mouse model of the pediatric brain tumor ependymoma that has permitted us to investigate Eph mediated cellular transformation.

Ependymomas are the third most common pediatric CNS malignancy, accounting for 6–12% of brain tumors in children with a mean age of diagnosis of 4 years (25–40% of patients < 2 years), a 5-year survival rate ranging from 39%–64% and a progression free survival rate of 23–45%^22^,^23^,^24^,^25^,^26^. Due to their chemoresistant nature, surgery followed by radiation is the most effective treatment option; unfortunately, despite advances in radiotherapy, cerebral radiation treatment of younger children (<3 years) has associated risks due to the heightened sensitivity of the developing brain. Therefore, better adjuvant treatments are needed, especially for this patient population^27^,^28^,^29^,^30^,^31^. Given the extremely limited number of ependymoma model systems to study the disease, we developed our current mouse model system as a tool to identify the key genes and pathways deregulated in disease development, growth and survival.

Previous ependymoma studies relied on orthotropic or flank human xenograft models even though they are extremely difficult to derive and maintain (*in vivo* and *in vitro*) because ependymoma cell lines did not exist^32^,^33^,^34^. We developed a highly reproducible intracranial implantation mouse model of the disease that overcomes these barriers, making it ideal for molecular and genomic experimentation^35^. To create this model, first 204 patient samples were analyzed for copy number variations via SNP array and 83 of these samples by gene expression profiling, which identified two mutational events (deletion of the INK4A/ARF locus and amplification/overexpression of the ephrin-B receptor EphB2) as putative supratentorial ependymoma tumor suppressor (INK4A/ARF locus) and oncogene (EphB2). Their role in ependymoma development was validated by overexpressing EphB2 in supratentorial E14.5 neural stem cells (STeNSCs) (previously identified as the potential cell of origin for ependymoma^36^) deleted of the Ink4a/ARF locus and implanting these cells in recipient mice brains proximal to the third ventricle. The implanted cells were transformed 4–8 months post-implantation (203 days median time of tumor development) into tumors that are histologically and molecularly similar to the human supratentorial disease in 100% of mice.

Our mouse model clearly identifies EphB2 as an ependymoma oncogene and a requisite for cellular transformation; however, the mechanism underlying this function remains unknown. The current study demonstrates that in addition to protein overexpression, receptor-mediated forward signaling is required for transformation while reverse signaling is not. Furthermore, transformation sensitizes the cell to ligand-induced differentiation inhibition, increased neurosphere formation and altered pathway signaling predominantly through phosphorylation changes.

## Results

### EphB2 activation is required for transformation

**Figure 1:**
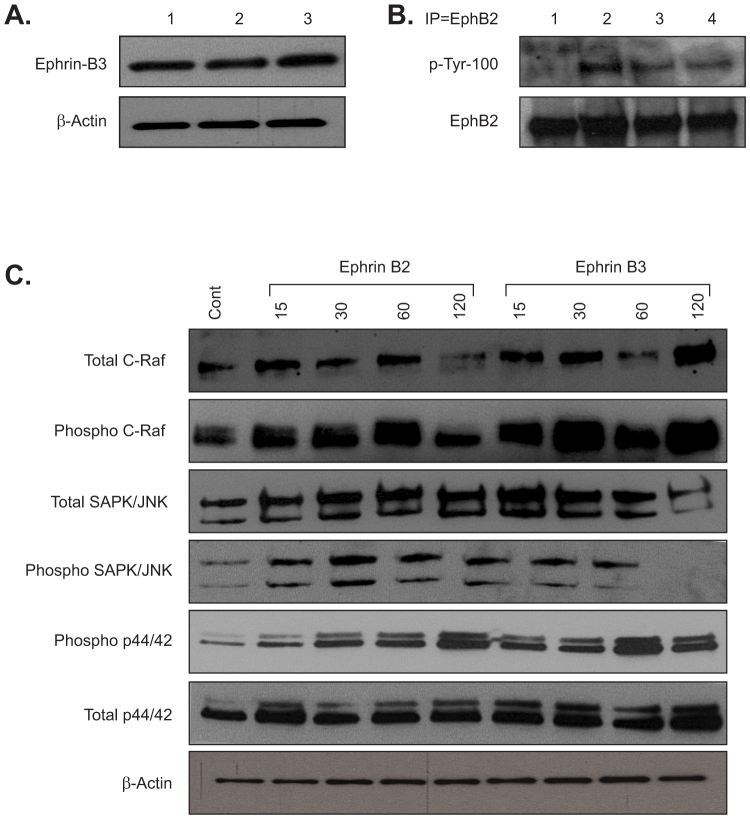
Exogenous but not endogenous ephrin-B ligand can activate EphB2 in EphB2 overexpressing Ink4a/Arf^(−/−)^ STeNSCs (utNSCs). (a) Ephrin-B3 immunoblot of uninfected Ink4a/Arf^(−/−)^ STeNSCs (lane 3) or infected with control (pCX4-RedEX; lane 1) or EphB2 expressing (lane 2) retrovirus. (b) *In vitro* EphB2 activation assay of utNSCs incubated for 24 hrs. with anti-human-IgG (lane1), ephrin-B1 (lane 2), ephrin-B2 (lane 3), or ephrin-B3 (lane 4). (c) Immunoblot of phospho- and total C-Raf, SAPK/JNK and p44/42 of utNSCs incubated with anti-human-IgG (lane1), and ephrin-B2 or ephrin-B3 for 15, 30, 60, and 120 minutes.

A key component of this model is the ability to grow EphB2 overexpressing Ink4a/Arf^(−/−)^ STeNSCs, henceforth called utNSCs, *in vitro* as tight compact balls called neurospheres. However, these cells are obtained from the subventricular zone of the embryonic mouse brain a region known to express all three ephrin-B ligands therefore it was necessary to determine if cultured utNSCs expressed ephrin-B ligand as well^37^,^38^,^39^. Western blot analysis illustrates the expression of ephrin-B3 in cultured utNSCs (Figure 1a); unfortunately, we were unable to definitively demonstrate ephrin-B1 or ephrin-B2 expression by western blot (Supplementary Figure 1a). An EphB2 receptor activation assay was performed on utNSC by immunoprecipitating (IP) for EphB2 then western blotting for phospho-tyrosine (p-Try) and clearly shows EphB2 in its inactive (unphosphorylated) rather then active (phosphorylated) state (Figure 1b, lane 1). To ensure that our overexpressed receptor was capable of ephrin-B ligand mediated activation, utNSCs were incubated with clustered ephrin-B(1-3)-Fc chimeric protein or human-IgG-Fc (negative control) (*in vitro* activation assay) prior to assaying for receptor activity. As the assay clearly demonstrates, utNSCs incubated with any of the three ephrin-B ligands (ephrin-B1-Fc, ephrin-B2-Fc and ephrin-B3-Fc) show ligand specific EphB2 phosphorylation as control treated cells (human-IgG-Fc) show no phosphorylated receptor (Figure 1b, lanes 2–4)). Additionally, EphB2 activation is titratable and dependent on ligand concentration and receptor phosphorylation is detectable 48 hrs. post ligand addition (Supplemental Figure 1b & 1c); however, the ramifications of this sustained phosphorylation is unknown. Ligand-specific EphB2-mediated signaling was confirmed by the ligand-induced rapid phosphorylation of the established downstream ephrin signaling targets Erk1/2 (p44/42), SAPK/JNK and C-Raf (Figure 1C).

**Figure 2:**
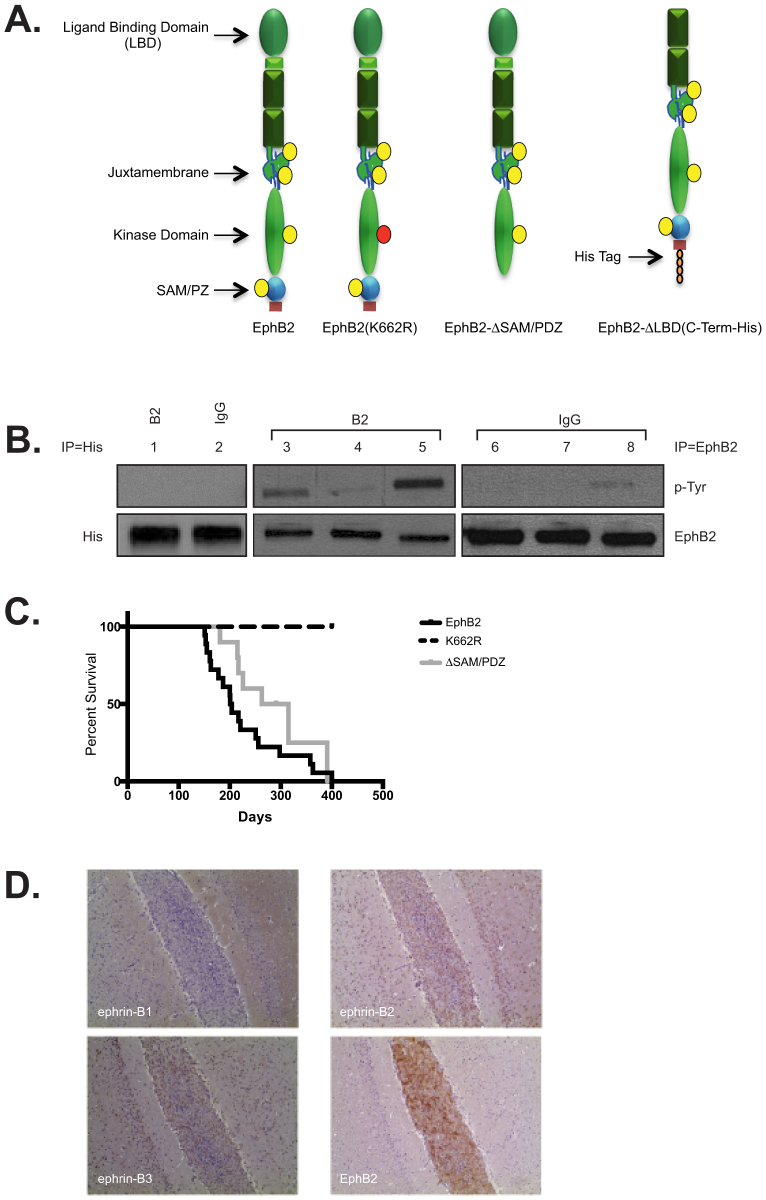
EphB2 activation is required for utNSCs transformation. (a) Structure of EphB2 mutant and domain deletion receptors. Yellow circle = Established phosphorylation site; Red circle = K662R mutation. (b) *In vitro* receptor activation assay of Ink4a/Arf^(−/−)^ STeNSCs infected with retrovirus expressing EphB2 (lanes 3 and 6), EphB2- ΔLBD(C-Term-His) (lanes 1 and 2), EphB2(K662R) (lanes 4 and 7) or EphB2-ΔSAM/PDZ (lanes 5 and 8) incubated with ephrin-B2 (B2 lanes 1, 3–5) or anti-human-IgG (IgG lanes 2, 6–8). Lanes 1 and 2 were immunoprecipitated and immunoblotted for EphB2- ΔLBD(C-Term-His) using a His specific antibody. Lanes 3-8 were immunoprecipitated for EphB2 and immunoblotted for phospho-tyrosine (p-Tyr; top) or EphB2 (bottom). (c) Survival curve of mice intracranially implanted with Ink4a/Arf^(−/−)^ STeNSCs retrovirally infected with EphB2 (n = 18; black), EphB2(K662R) (n = 7; dash) or EphB2-ΔSAM/PDZ (n = 10; grey). (d) Ephrin-B(1-3) and EphB2 immunohistochemical staining of mouse brain intracranially implanted with tdNSCs.

An array of EphB2 mutants were used to evaluate the role of receptor forward and reverse signaling on tumor formation (Figure 2a): 1) EphB2(K662R)—kinase domain inactivated and inhibited in forward signaling but retaining ephrin-mediated reverse signaling^19^. 2) EphB2(ΔSAM/PDZ)—deleted intracellular carboxy-terminal SAM/PDZ domain; while less studied, phosphorylation of the SAM/PDZ domain is involved in receptor self-association and protein-protein interactions^5^,^6^ and 3) EphB2-ΔLBD(C-Term-His)—with a carboxy-terminal 6XHis tag (required for protein detection) and deleted extracellular ligand-binding domain (LBD) to inhibit ligand binding resulting in loss of forward and reverse signaling. All mutants were found to express equivalent levels of protein when the same MOI was used (data not shown). *In vitro* activation assays were used to evaluate each mutant (Figure 2b). As expected, EphB2 (lanes 3 and 6) and EphB2(ΔSAM/PDZ) (lanes 5 and 8) were activated by ephrin-B2 (B2 lanes) ligand while EphB2(K662R) (lanes 4 and 7) and EphB2-ΔLBD(C-Term-His) (lanes 1 and 2) remain largely unaffected by the addition of ligand (B2 lanes). Our receptor activation assay did uncover very minimal phosphorylation of EphB2(K662R) in the presence of ligand; however, this had no effect on any functional assays performed in this study. The C-term-His tag was placed on the carboxy-terminus of our EphB2-ΔLBD protein due to our inability to find an EphB2-specific antibody that could recognize this mutant protein. As a result a His-Tag specific antibody replaced the EphB2 antibody for the *in vitro* activation assay of EphB2-ΔLBD(C-Term-His).

Next, the transformational potential of each mutant was ascertained by examining the implanted mouse survival curves and whether they had a tumor upon death (Figure 2c). Similar to our previous study, wild-type EphB2 (black) was capable of cellular transformation and tumor formation in 100% of implanted mice (18/18) with an median survival of 203 days ranging from 151 to experiment termination at day 400. Interestingly, the SAM/PDZ domain was discovered to be dispensable for transformation as all implanted animals formed tumors when EphB2(ΔSAM/PDZ) (grey) was overexpressed (8/8). Even though the survival curve of EphB2(ΔSAM/PDZ) implants appears to have a longer tumor latency than wild-type EphB2, it was not statistically significant (203 vs. 289 day median survival for EphB2 vs. EphB2(ΔSAM/PDZ) respectively). However, tumor formation was not seen when the inactive EphB2(K662R) (7/7) mutant (dash line) and EphB2-ΔLBD(C-Term-His) (data not shown; 8/8) receptors were overexpressed, further support for the requirement of receptor activation in tumor formation. Given the requirement for receptor activation in transformation, it needed to be determined if the conventional mechanism of EphB/ephrin-B signaling was involved. Since cells expressing receptor and those expressing ligand require cell-cell contact to initiate activation, it was necessary to examine the ephrin-B expression profile at the implantation site (Figure 2d). EphB2-driven mouse tumor cells were implanted intracranially into recipient mice at the same coordinates used to create our model. Animals were sacrificed 48 hrs post implantation and stained for EphB2 (to identify location of implanted cells and confirming the implantation site), ephrin-B1, ephrin-B2, and ephrin-B3. Ephrin-B3 and ephrin-B2 were expressed unmistakably on neuronal cells at the implantation site, suggesting that a classic mechanism is most likely involved in EphB2 activation leading to transformation.

**Figure 3:**
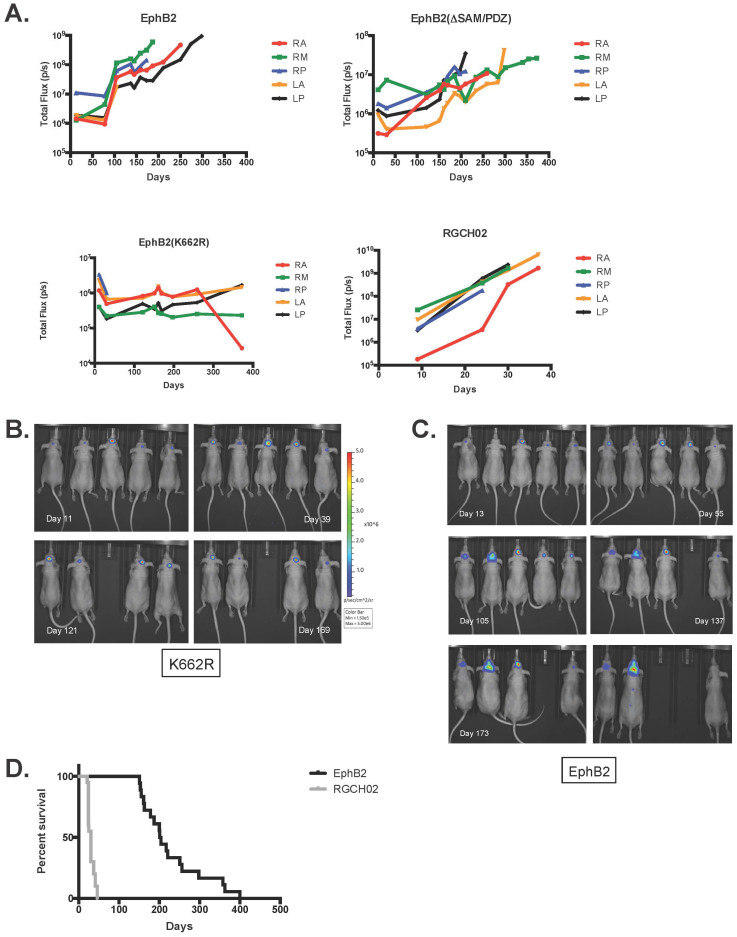
Luciferase activity of mice IC implanted with various EphB2 overexpressing Ink4a/Arf^(−/−)^ STeNSCs. (a) Graph of total luminescence (p/s) over time (days) for IC implanted Ink4a/Arf^(−/−)^ STeNSCs retrovirally infected with MSCV-Luc (luciferase expressing retrovirus) and either EphB2 (top left), EphB2(K662R) (bottom left) or EphB2-ΔSAM/PDZ (top right). tdNSCs represents mice IC implanted with the tdNSC line RGCH02 (bottom right). Bioluminescence images of EphB2(K662R) (b) and EphB2 (c) IC implanted mice over time (days). (d) Survival curve of mice IC implanted with 1 × 10^6^ utNSCs (n = 18; black) or the tdNSC line RGCH02 (n = 20; grey). **** (p < 0.0001) vs. EphB2.

Two possibilities could account for the inability of EphB2(K662R) and EphB2-ΔLBD(C-Term-His) infected Ink4a/Arf^(−/−)^ STeNSCs to form tumors: First, the implanted cells remain viable but are unable to grow intracranially. Alternatively, implanted cells are incapable of surviving in the brain in the absence of EphB2 expression and die post implantation. To determine which scenario was correct utNSCs were infected with a luciferase expressing retrovirus (MSCV-Luc-IRES-YFP) to monitor and measure luciferase activity as an indicator of cell viability and growth (Figure 3a). All implant mice regardless of EphB2 retrovirus used to infect them show a relatively stable baseline luciferase activity ranging from approximately 5 × 10^5^ to 5 × 10^6^ (pixels (p)/s) indicating that some percentage of cells remain viable indefinitely post-implantation (Supplemental Figure 2). It is clear from the luminescence data that implanted cells remain viable and quiescent as the luciferase signal from EphB2(K662R) infected cells is detectable and stable for months post-implantation (Figure 3b). Surprisingly, while EphB2 overexpressing cells have a similar profile for a minimum of two months post-implantation, a sharp increase in signal intensity and change in animal phenotype (domed skull, hunched posture, thin appearance and lethargy) always follows, indicating tumor growth (Figure 3c). All animals sacrificed at this point contain tumor when examined. The similar sustained and constant luciferase signal we observe for control and EphB2 implantation mice is suggestive of a viable untransformed cell. The sudden, rapid and sustained increase in signal seen in EphB2-implanted mice is indicative of transformed cells undergoing rapid growth. The most probable reason for this profile is that before this expansion, cells are acquiring the necessary additional mutations required for transformation into ependymoma. Interestingly, when a similar number of the EphB2-driven mouse primary ependymoma tumor cells (tdNSCs) RGCH02 were intracranially implanted at our current site, we see a very different luminescence profile (Figure 3a; RGCH02) and survival curve (Figure 3d). Unlike the untransformed luminescence curve previously described, there is not a lengthy sustained signal but rather the classic rapid signal increase indicative of cellular growth followed by animals succumbing to tumor formation much quicker leading to a decreased in survival time (30 day median survival). This would strongly suggest that unlike untransformed utNSCs, transformed utNSCs are capable of rapid growth *in vivo* further supporting the hypothesis that additional changes along with receptor overexpression and activation are vital for EphB2-mediated cellular transformation.

### EphB2-driven mouse tumorspheres (tdNSCs) are distinct from their EphB2 overexpressing untransformed (utNSCs) counterparts

**Figure 4:**
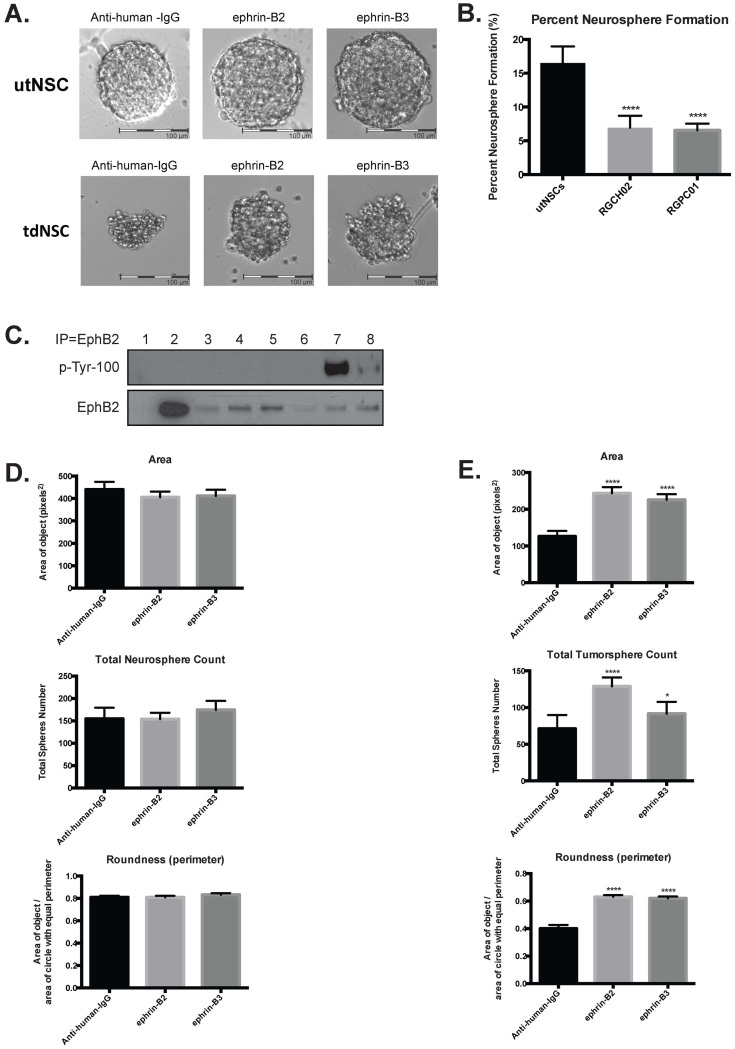
EphB2 driven tumorspheres are uniquely responsive to ephrin-B ligand. (a) Phase contrast image (20×) of utNSCs (top) and tdNSCs (bottom) after 6-day incubation with anti-human-IgG, ephrin-B2 or ephrin-B3. (b) utNSCs, RGCH02 and RGPC01 sphere formation assay (**** (p < 0.0001) vs. utNSCs). (c) IP/Western of Ink4a/Arf^(−/−)^ STeNSCs (lane 1), utNSCs (lanes 2), tdNSC lines RGPC02, RGCH01-RA, RGPC01-RP, and RGPC03-RM (lanes 3–6), utNSCs treated with ephrin-B2 (lane 7) or anti-human-IgG (lane 8). IP with EphB2 antibody, western blot with phospho-tyrosine (top) or EphB2 (bottom). Graphs of neurosphere (d) and tumorsphere (e) number, area and roundness after treatment with anti-human-IgG, ephrin-B2 or ephrin-B3. * (p < 0.05) and **** (p < 0.0001) vs. anti-human-IgG.

Similar to Ink4a/Arf^(−/−)^ STeNSCs and utNSCs the EphB2 driven mouse tumors (tdNSC) grow *in vitro* in stem cell conditioned media as tumorspheres; however, cultured tdNSCs and utNSCs differ in several pronounced ways: 1) Morphologically (Figure 4a; anti-human-IgG): utNSCs form classical smooth, shine, symmetrical neurospheres with a clear outer ‘shell’ and a dark center. Tumorspheres derived from tdNSCs are dark and frayed around the edges with a disorganized appearance. Additionally, when roundness is measured (measure of how spherical an object is with a perfect sphere having a value of 1.0) utNSCs have an average roundness score of 0.81 verses 0.399 for tdNSCs. 2) Neurosphere forming potential: Although both utNSCs and tdNSCs form spheres, they form them at significantly different rates; utNSCs have the greater capacity (16.51% for EphB2-overexpressing eNSCs vs. 6.9% and 6.5% for the tdNSC lines RGCH02 and RGPC01 respectively) (Figure 4b). 3) Sphere growth rate as determined by area: In addition to producing more neurospheres, the growth rate (increase in size) of the spheres is significantly higher for utNSCs than tdNSCs as manifested by the larger sphere size when measured six days post single cell plating (area of 440 p^2^ for utNSCs compared to 126 p^2^ for RGCH02). There is, however, one very striking similarity between tdNSCs and utNSCs, both express EphB2 in its inactive form (Figure 4c).

The expression of ephrin-B3 and ephrin-B2 at the implantation site strongly implicates them in EphB2-mediated transformation therefore we examined their effects on utNSC and tdNSC sphere morphology, size and sphere formation potential. It was clear that tdNSCs were significantly different from utNSCs in sphere morphology, size, and shape (roundness) in the absence of ligand exposure and EphB2 activation. We wanted to learn if any of these characteristics are altered by ephrin-B ligand-mediated EphB2 activation and if so is there a difference between the ligands. tdNSCs and utNSCs cells were exposed to clustered anti-human IgG (negative control), ephrin-B2-Fc or ephrin-B3-Fc for 6 days and analyzed microscopically (Figure 4a) and measured for sphere size (area) and roundness (Figure 4d & 4e). Interestingly, we find that utNSCs (Figure 4d) show no significant change in any of these parameters displaying an insensitivity to short term EphB2 activation (<9 days; represents three or fewer ligand administrations). This is not the case for tdNSCs that exhibit an increase in total tumorsphere number with the addition of both ligands (from 71 tumorspheres with anti-human IgG to 128 and 91 tumorspheres with ephrin-B2 and ephrin-B3 respectively) (Figure 4e) accompanied by quantifiable changes in tumorsphere size and shape. The most obvious is the increase in tumorsphere size from an average of 126.5 pixels(p)^2^ (Anti-human IgG-Fc) to 243.5 p^2^ and 225.6 p^2^ in the presence of ephrin-B2 and ephrin-B3 respectively. Unlike the unchanged roundness score of 0.81 of utNSCs regardless of ligand administration; the roundness score of tdNSCs (roundness score of 0.399) is altered drastically by the addition of ephrin-B2 (roundness score of 0.63) or ephrin-B3 (roundness score of 0.62).

**Figure 5:**
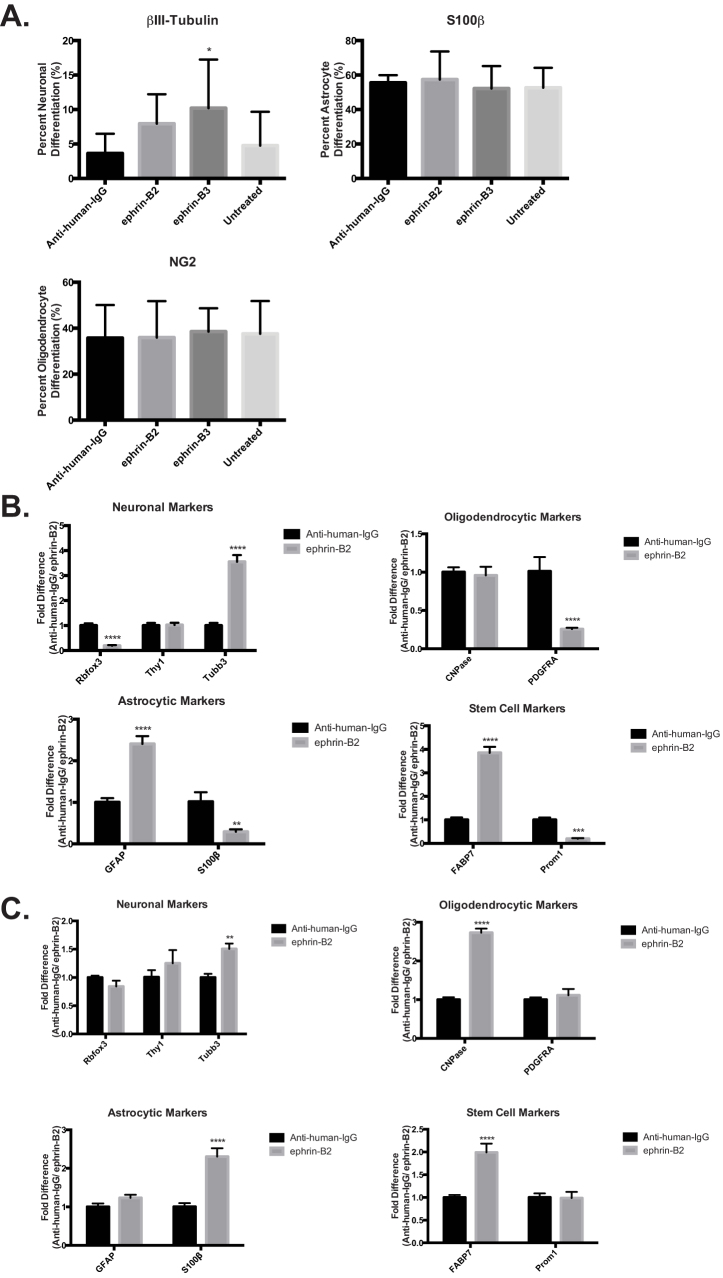
Ephrin-B ligand exposure specifically inhibits EphB2 driven tumor cell differentiation. (a) Graph depicting the percent of utNSCs differentiated into astrocyte (βIII-tubulin), neuron (S100) or oligodendrocytes (NG2) after treatment with nothing (untreated), anti-human-IgG, ephrin-B2 or ephrin-B3. Neuronal type determined by immunofluorescent staining for neuronal lineage specific marker and cellular morphology. qPCR analysis of differentiation assay of tdNSCs (b) and utNSCs (c) treated with anti-human-IgG or ephrin-B2. All qPCR values are normalized to GAPDH. qPCR primer sets: Rbfox3 (NeuN), Thy1, Tubb3 (βIII-Tubulin), CNPase, PDGFRA, GFAP, S100β, FABP7 (Blbp), Prom1 (CD133). * (p < 0.05), ** (p < 0.01), *** (p < 0.005), and **** (p < 0.0001) vs. anti-human-IgG.

The sensitivity of our tumor line to ephrin ligand and the changes induced by EphB2 activation uncovered the possibility that receptor activation may affect other tdNSC traits. We know our tdNSCs originate from eNSCs with the ability to differentiate into the three neuronal lineages (astrocytes, neurons and oligodendrocytes). To determine if and to what extent this characteristic is maintained in tdNSCs and the role EphB2 activation may play in both utNSC and tdNSC differentiation, we used our differentiation assay. The assay consists of growing tdNSCs or utNSCs in 1% serum, to enhance any differences, for 7 days, to induce differentiation in the presence of human IgG-Fc (negative control), ephrin-B2-Fc or ephrin-B3-Fc. Neuronal lineage was determined by simultaneously assessing cells for positive immunofluorescence staining (IF) with a lineage specific marker (astrocytes (S100β), oligodendrocyte (NG2), neuron (βIII-Tubulin)) and by visual verification of correct cellular morphology. Like the neurosphere experiments, utNSC were unaffected by the addition of ligand (Figure 5a) differentiating into predominantly astrocytes (≈55%) followed by oligodendrocytes (≈35%) and neurons (≈3.66%). We did observe a slight but significant increase in neuronal differentiation with ephrin-B3 but due to the high degree of variation we are unsure of its meaning. Further validation of these results can been found in the similar differentiation profile and ligand insensitivity of Ink4a/Arf^(−/−)^ STeNSCs and EphB2(K662R)-overexpressing Ink4a/Arf^(−/−)^ STeNSCs in comparable assays (Supplemental Figure 3).

Previous experience has shown that lineage markers can be expressed at various stages in differentiation irrespective of cellular morphology; therefore the combination of IF and morphology was found to be the most accurate assessment of neural differentiation. Unfortunately, tdNSCs express both GFP and RFP making this method of assessment impossible and driving the use of quantitative real-time PCR (qPCR) using neuronal lineage specific primers as an alternate method. qPCR illustrates that ephrin-B ligand had a profound effect on tdNSC differentiation resulting in increased (βIII-Tubulin (Tubb3), GFAP and FABP7) and decreased (PDGFRα, NeuN (Rbfox3), and S100β) marker expression (Figure 5b). Detailed analyses of the expression changes and the differentiation stage each marker represents, elucidates a clearer understanding of receptor activation. In all cases of differentiation (oligodendrocytic, neuronal and astrocytic) receptor activation in the tumor cell line caused either an increase in the expression of markers representing a less differentiated state (i.e. NCam1, βIII-Tubulin and GFAP) or a decrease or no change in markers representing a more differentiated state (i.e. S100β, PDGFRα and NeuN). Most striking is an increase in the radial glial cell/stem cell marker FABP7 (aka Blbp) further supporting the hypothesis that EphB2 activation induces a more stem-like state. A similar qPCR analysis was done on ephrin-B ligand treated utNSCs to examine how similar analysis of a differentiation assays by IF or qPCR would be. Interestingly, unlike our IF experiment, an effect of ephrin-B ligand on utNSCs differentiation is detectable (Figure 5c). Receptor activation in utNSCs elevates expression of markers indicative of more differentiated oligodendrocytes (CNPase) and astrocytes (S100β) and early stage neuronal markers (NCam1, βIII-Tubulin). It is intriguing to note that receptor activation did not reduce expression of any of the markers studied in utNSCs. Once again, tumor cell responsiveness to ligand addition and receptor activation results in apparent conversions to a less differentiated, more stem-like state is revealed.

### Signaling pathway evaluation

**Figure 6:**
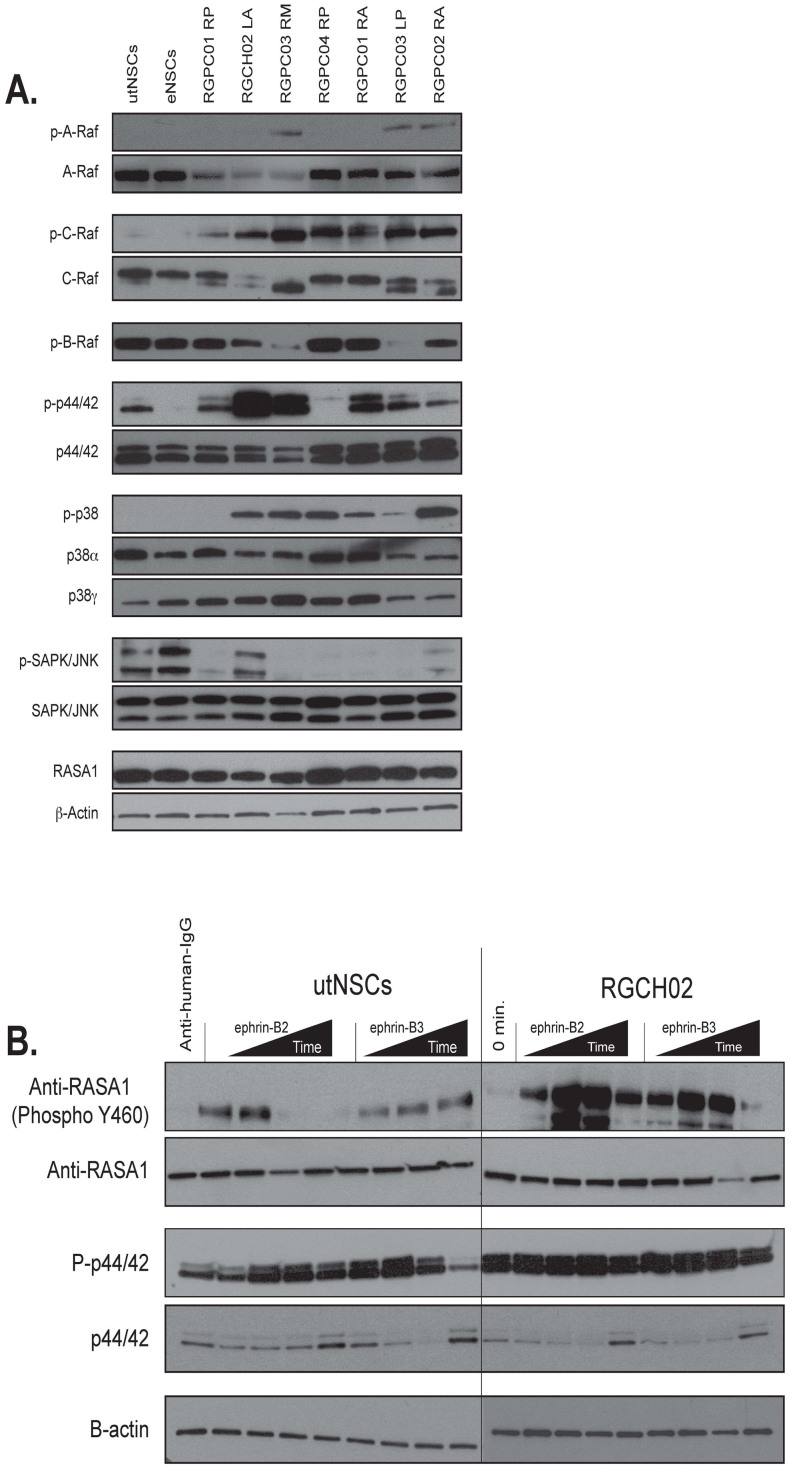
EphB2 driven tumor cell lines exhibit a different phosphoprotein expression pattern then utNSCs independent of receptor activation. (a) Western blot analysis of total and phosphorylated protein expression in utNSCs, Ink4a/Arf^(−/−)^ STeNSCs (lane labeled eNSC) and seven tdNSC lines for signaling proteins involved in Ras (A-Raf, B-Raf, C-Raf, Mek1/2, and p44/42, RASA1), p38 MAPK (p38α and p38γ) and SAPK/JNK. Western blot analysis of p44/42 (b) and RASA1 (c) phosphorylation after *in vitro* utNSCs or RGCH02 initial EphB2 activation with anti-human-IgG (negative control) or 15, 30, 60, and 120 minute treatment with either ephrin-B2 or ephrin-B3.

Since the predominant outcome of ephrin receptor activation is kinase-mediated phosphorylation of critical regulatory proteins, we surmised that aberrant ligand-independent phosphorylation changes might highlight pathways crucial for transformation. Although we are unable to assay these pathways *in vivo*, our model affords us the unique ability to compare a normal cell (utNSC) and its direct transformed counterpart (tdNSC) for such differences. Using western blot analysis, we examined the expression levels and phosphorylation state of the Ras-Raf, p38 MAPK and SAPK/JNK pathways in Ink4a/Arf^(−/−)^ eNSCs, utNSCs and seven independently derived tdNSC (Figure 6a). The Ink4a/Arf^(−/−)^ eNSCs were used to establish the normal protein expression levels and phosphorylation state and the utNSCs to determine the effects of overexpressing EphB2 in Ink4a/Arf^(−/−)^ eNSCs in the absence of transformation. Due to the number and nature of our tumor samples, any change observed in at least 70% (5 of 7) of tumor samples when compared to control samples were considered significant. With the exception of p44/42, there were no discernible significant differences in the protein levels or phosphorylation states between Ink4a/Arf^(−/−)^ eNSCs and utNSCs suggesting that, in the absence of activation, EphB2 overexpression has no effect on these particular pathways. Similarly, few differences in protein levels were observed when comparing utNSCs to tdNSCs, indicating that the involvement of these signaling pathways in transformation is not due to aberrant protein expression. In contrast, definitive differences between utNSCs and tdNSCs can be seen when comparing the phosphorylation state of these same proteins; the most striking being the conversion of both C-Raf, p38 and p44/42 from an unphosphorylated to phosphorylated state in 7/7, 6/7 and 5/7 tdNSC lines respectively. We also observed decreased phosphorylation of SAPK/JNK (6/7) in our tumor lines relative to utNSCs. These phosphorylation changes are specific as we observe an increase in A-Raf phosphorylation in only 3/7 tdNSC lines. We also observe decreased B-Raf phosphorylation in 2/7 tumor derived cell lines; however total B-Raf was undetectable via western blot so nothing definitive can be deduced. These results imply that post-translational modifications as opposed to protein level changes are the principle mechanism underlying EphB2-mediated transformation.

Thus far, the data demonstrates a unique sensitivity of the tdNSCs to EphB2 activation that is not shared by utNSCs. Additionally, the prevalent difference between these two cell types resides in the phosphorylation state of key proteins involved in EphB2 signaling. Given these results, we wanted to ascertain if this increased sensitivity correlates to changes in EphB2-mediated protein phosphorylation. An initial ephrin-B2 and ephrin-B3 activation experiment was performed with utNSCs and RGCH02 tdNSCs and assayed for phospho-p44/42 (increased phosphorylation in RGCH02) and phospho-RASA1 (unphosphorylated in both cell lines) expression (Figure 6b & C). Comparable to previous data, the tumor cell line is more responsive to ligand administration, displaying a robust increase in phosphorylation in both cases that is sustained markedly for p44/42. Although only two proteins were tested, it is conceivable that similar heighted responses could represent an additional required cellular response to continual receptor activation necessary for tumor formation.

## Discussion

The role of EphB2 as an ependymoma oncogene is demonstrated clearly in our implantation mouse model of the disease; however, the requirement and potential impact of receptor activation on this process is unknown. The current study shows that EphB2 overexpression alone is insufficient for Ink4a/Arf^(−/−)^ STeNSC transformation; receptor activation and acquisition of additional alterations over time are key elements as well. It is improbable that transformation is a result of transient receptor activation given the rapidity of EphB2 mediated signaling and our inability to detect any significant changes in proliferation, differentiation or stem cell marker expression in utNSCs after a 6-day exposure to ligand. More likely, the necessary changes are acquired in response to sustained EphB2 activation. One of the greatest strengths of our system is the ability to culture, expand and directly compare utNSCs to their transformed counterpart (tdNSCs). Comparisons revealed a significant difference between the cell types regarding changes in protein phosphorylation state rather than expression level, supporting the possibility that the additional elements cooperating with EphB2 activation in cellular transformation are post-translational in nature. While this study focused on established ephrin-mediated signaling pathways, similar alterations in other pathways may contribute as well. Currently, we are investigating the *in vitro* effects of prolonged (>14 days) ligand exposure and receptor activation in an attempt to recapitulate our *in vivo* implantation data.

The ligand-mediated cis-inhibition we observe *in vitro* strongly suggests that the ephrin receptor and ligand expression pattern of cells adjacent to utNSCs have a profound impact on tumor formation. Despite the fact that reverse signaling is not involved in EphB2 mediated transformation, it may be a cis-inhibitor of EphB2 forward signaling. The ephrin-B3 ligand western blot and *in vitro* activation assay of utNSCs shows that endogenously expressed ligand is incapable of activating EphB2 but exogenous ligand completely retains its receptor activation capability; this clearly indicates the cis-inhibitory effects of ligand mediated reverse signaling as this function is the only distinct difference between the two ligands. Furthermore, recent data^40^,^41^ have revealed such an effect when receptor and ligand are activated within the same cell. This type of feedback loop provides an additional layer of regulation to control where and when transformation occurs, which, in essence, ensures that only cells that overexpress EphB2 and are adjacent to cells expressing ligand but not receptor can undergo EphB2-mediated transformation.

Additional constraints on where and when ependymomas develop may be a function of changes in EphB2 and ephrin-B ligand expression levels and patterns during normal CNS development. Studies in mice and rats have demonstrated ephrin receptor and ligand expression vary in both pattern and levels over the course of CNS formation, being highest early in CNS maturation and diminishing as development progresses until their comparative absence in adulthood^37^,^38^,^39^. Since EphB2 is expressed in eNSCs, it is plausible that the increased EphB2 expression observed in ependymoma is the result of the cells inability to shutoff receptor expression. In adulthood, EphB2 levels are lost while ephrin-B ligand expression is highly restricted to regions of active neurogenesis such as the subventricular zone (SVZ) and subgranular zone of the hippocampus (SGZ)^37^,^38^,^39^. In particular, expression of ephrin-B1 and ephrin-B2 has been noted in stem/progenitor cells within the neuroepithelium of the lateral ventricle and SVZ of neonatal mice and adult mice, albeit at lower levels; contrarily, ephrin-B3 expression was detected outside of this region^37^,^38^,^39^,^42^. Thus, a cell that became capable of maintaining EphB2 expression (e.g. chromosomal amplification, deregulated transcription) would be in a perfect location to support extensive ligand/receptor interactions.

Although the exact mechanism underlying EphB2-mediated ependymoma development remains unclear, the response of untransformed and transformed eNSCs to ligand mediated ephrin signaling may shed light on this mystery. First, the difference in response of utNSCs (unresponsive) and tdNSCs (responsive) to ligand indicates that sensitivity to EphB2/ephrin-B mediated signaling is acquired by the cells. More importantly, they highlight a potential avenue for tumor formation involving retention of a neural stem cell state upon EphB2/ephrin-B activation that ultimately results in cellular transformation. Whether this sensitivity is a result of or a prelude to transformation cannot be determined by this study, but based on previous studies by others, we believe it to independent of transformation. Cells within the adult mouse and rat SVZ are responsive to ephrin signaling independent of this process^42^,^43^,^44^,^45^. Interestingly, these studies showed an increase in adult NSC proliferation and a decrease in neurogenesis by ephrin signaling inhibition, results that are not only opposite to the unresponsiveness seen in utNSCs and EphB2 non-expressing Ink4a/Arf^(−/−)^ STeNSCs but also correlate with the response seen in tdNSCs upon activation of ephrin signaling. Consequently, this difference may explain the inability of EphB2 overexpressing adult Ink4a/Arf^(−/−)^ STeNSCs to develop into ependymoma when used in our implantation system.

**Figure 7:**
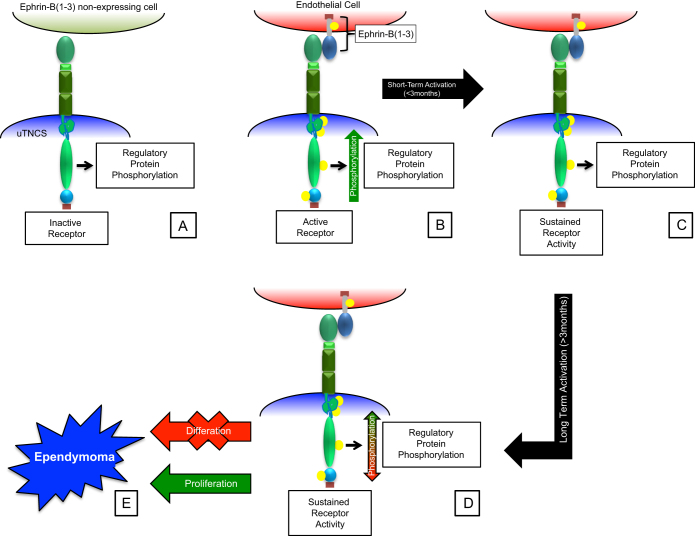
Schematic diagram of proposed model of EphB2 mediated ependymoma development. (A) utNSCs within an ephrin-B ligand free microenvironment. (B) utNSCs within an ephrin-B ligand expressing microenvironment resulting in EphB2 activation and normal regulated ephrin signaling pathway activation. (C) Sustained EphB2 activation (<3months) but no accompanying change in regulatory protein phosphorylation (similar phosphorylation pattern as (A)). (D) Sustained EphB2 activation (>3months) with accompanying change in regulatory protein phosphorylation pattern (differing phosphorylation pattern from (A)). (E) Transformation into tdNSC as evident by inhibited differentiation and increased proliferation.

From our data we propose the following model for EphB2 mediated ependymoma development (Figure 7): 1) sustained utNSC ephrin signaling as a result of the interaction of EphB2 (expressed by utNSCs) with adjacent ephrin-B ligand expressing ephrin receptor non-expressing cells within the SVZ; 2) inhibition of utNSCs differentiation; 3) increase in utNSC proliferation; 4) stable alterations in the phosphorylation state of key regulatory signaling protein resulting in sustained changes in signaling pathway activity (i.e. Ras and MAPK) and 5) induction of angiogenesis via forward and reverse ephrin signaling between utNSC and endothelial cells within the microenvironment. Currently, we are refining our model, identifying the key signaling pathways, and determining the order of events. In addition to ependymoma, other gliomas, such as glioblastoma multiforme (GBM), overexpress EphB2; it would be interesting to ascertain if a similar mechanism of EphB2-mediated transformation is involved in their development as well. If so, this would substantiate the oncogenic power of EphB2.

## Methods

### Cell culture

Supratentorial embryonic neural stem cells and tumor cells were prepared, cultured as neurospheres and infected with appropriate retrovirus (MOI = 1) as previously described^34^. Neural stem cells and tumor cells were maintained in complete neural basal media containing 1× N2, 1× B27, Pen/Strep, L-glutamine, bFGF, EGF and BSA (Sigma-Aldrich, St. Louis, MO) and grown at 37°C in 5% CO^2^. All tissue culture materials were obtained from Life Technologies, Grand Island, NY unless otherwise stated.

### Retroviral production

Retrovirus produced as previously described^34^. In short, 293T cells were obtained from Dr. Richard Gilbertson (St. Jude Children's Research Hospital, Memphis, TN) grown and maintained at 37°C with 5%CO^2^ in DMEM media (w/4.5 g/L Glucose; Bio Whittaker) in L-Glut, Pen/Strep and 10%FBS. 3 × 10^6^ cells/10 cm^2^ dish plated and grown overnight. Media replaced with complete neural basal media prior to transfection. 3 μg of VSV-ENV and GAG-pol and 8.5 μg of EphB2 vector used following FuGene6 transfection protocol as directed by company (Promega, San Luis Obispo, CA). Media containing virus collected every 12 hrs. post-transfected and replaced with fresh complete neural basal media.

### EphB2 mutant retroviral cloning

PCX4-EphB2-ΔLBD(C-term-His): The following primer pair was used to place a C-term His-tag on the carboxy-terminus of EphB2. (Primers: F: 5′- AGATAAATGGGAGCCCGGGTC -3′; R: 5′- CTA**GTGGTGATGGTGATGATG**AACCTCTACAGACTGGATC -3′ (Bold = 6 × His Tag)). PCR product was cloned into the pCR-2.1-TOPO vector (Life Technologies, Grand Island, NY), checked for proper orientation (pCR-2.1-TOPO-EphB2), and EphB2 insert excised and cloned into the pCX4-RedEX vector using BamHI and NotI.

PCX4-RFP-EphB2 (K662R): Site-directed mutagenesis was done on PCX4-RFP-EphB2 (Primers: Mut K662R F: 5′- GATCTTTGTAGCCATCAgGACCCTCAAGTCAGGAT -3′; Mut K662R R: 5′- ATCCTGACTTGAGGGTCcTGATGGCTACAAAGATC -3′ (lower case = mutated base).

PCX4-EphB2(ΔSAM/PDZ): pCDNA-EphB2 (generously donated by the Gilbertson Lab at ST. Jude Children's Research Hospital) was site-directed mutated to create an additional XhoI site at position 2790 within EphB2 (Primers: a2790c F: 5′- GGTGGATGAGTGGCTCGAGGCCATCAAGATG -3′; a2790c R: 5′- CAT CTTGATGGCCTCGAGCCACTCATCCACC -3′). Resulting plasmid was digested with XhoI, filled in and cut with BamHI (remove SAM domain). PCX4-EphB2-RFP was cut with NotI, filled in, and digested with BamHI (remove EphB2). EphB2(ΔSAM/PDZ) insert was then ligated to the PCX4-RFP backbone.

### *In vitro* EphB2 activation

Ephrin-B ligand clustering: ephrin-B(1-3)-Fc (R&D Systems, Minneapolis, MN) and anti-human-IgG-Fc (Jackson ImmunoResearch Laboratories, West Grove PA) antibodies were incubated for a minimum of 2 hrs at 4°C at a molar ratio of 1:10 (ephrin-B-Fc to anti-human-IgG). For all in vitro activation assays 1 μg/ml clustered ligand was used unless otherwise stated.

*In vitro* activation: Cells were plated at 1 × 10^6^ cells/10 cm^2^ dish and grown for 48 hrs in complete neural basal media at 37°C with 5%CO^2^ with clustered anti-human-IgG-Fc or ephrin-B(1-3)-Fc ligand. Fresh clustered anti-human-IgG-Fc or ephrin-B(1-3)-Fc ligand was added every 72 hrs.

### Intracranial Implantation

1 × 10^6^ cells/mouse were resuspended in 5 μl of Matrigel (BD Bioscience, San Jose, CA) and injected into the right caudate putamen of 6 to 8-week-old female CD1^nu/nu^ nude mice (Charles River, San Diego, CA) over 2 min using a 26-gauge Hamilton syringe. All mice were maintained under isoflurane gas anesthesia for the entire procedure. Animals were immediately euthanized if they displayed neurologic deficits or abnormal phenotypes such as hunched posture, domed skull or lethargy. All animals remaining after 400 days post implantation were euthanized. At the time of euthanasia brains were either prepared for tissue culture (see above) or fixed overnight with 10% formalin, paraffin embedded, cut into 5 μM tissue sections, and stained with H&E. All animal experiments were approved by the Nationwide Children's Hospital Institutional Animal Care and Use Committee.

### In Vivo Bioluminescence Imaging

Bioluminescence imaging studies were conducted using the Xenogen Ivis Spectrum (Caliper Life Sciences, Hopkinton, MA). Animals received an intraperitoneal injection of 4.5 μg Xenolight Rediject D-Luciferin (Caliper Life Sciences) and were continuously maintained under isoflurane gas anesthesia. Images were obtained 20 minutes after luciferin administration. The bioluminescence intensity was quantified using Living Image Software (version 3.1; Caliper). Signal intensity was quantified as the sum of detected photons per second within the region of interest. We consider any increase >50× first measurement as suggestive of cellular proliferation. Definitive cellular proliferation is established when two consecutive measurements of >100× first measurement are made.

### Immunoprecipitation (IP) and western blot analysis

Cells were pelleted, and whole cell protein extract (WCE) made using RIPA Buffer (Thermo Scientific, Rockford, IL) containing protease and phosphatase inhibitors (Thermo Scientific, Rockford, IL) and protein concentration determined using Bradford Protein assay (Thermo Scientific, Rockford, IL). IP assays were performed using 100 μg of WCE and anti-EphB2 antibody (R&D Systems, Minneapolis, MN) diluted 1:200. All western blots were done using the Bio Rad Mini-Protean electrophoresis system (Bio Rad, Hercules, CA). Tyrosine phosphorylation of EphB2 was determined by western blot using the following antibodies: phospho-tyrosine (1:1000; Cell Signaling Technology, Boston, MA) and EphB2 (1:200; R&D Systems, Minneapolis, MN).

All western blot analysis were performed using 40 μg/sample of WCE and probed with the following antibodies: A-Raf, P-A-Raf (Ser299), P-B-Raf (Ser445), C-Raf, P-C-Raf (Ser259), p44/42, P-p44/42 (Thr202/Tyr204), SAPK/JNK, P-SAPK/JNK (Thr183/Tyr185), P-p38 (Thr180/Tyr182), p38α, p38γ, β-Actin, His-Tag (1:1000; Cell Signaling Technology, Boston, MA), RASA1 (AbCam, Cambridge, MA), Anti-ephrin-B1 (1:50; R&D Systems, Minneapolis, MN), Anti-ephrin-B2 (1:50; R&D Systems, Minneapolis, MN), Anti-ephrin-B3 (1:50; AbCam, Cambridge, MA).

### Immunohistochemistry and immunofluorescence assays

Immunohistocheminstry and immunofluorescence procedures were performed on 5 μM tissue sections as previously described using the following antibodies^35^: Anti-EphB2 (1:200; R&D Systems, Minneapolis, MN), Anti-ephrin-B1 (1:50; R&D Systems, Minneapolis, MN), Anti-ephrin-B2 (1:50; R&D Systems, Minneapolis, MN), Anti-ephrin-B3 (1:50; AbCam, Cambridge, MA), S100β (DAKO, Carpinteria, CA), NG2 and βIII-Tubuline (EMD Millipore, Darmstadt, Germany).

### Neurosphere Assay

Neurosphere formation assays were performed as previously described^34^. Neurosphere image acquisition, count, size and area determination was performed using the Leica DMI 4000B microscope (Leica Microsystems, Wetzlar, Germany) with Q Imaging Retiga-SRV Fast 1394 camera (Q Imaging, Surrey, Canada) and Surveyor 7.0.0.9 (Objective Imaging, Kansasville, WI) imaging acquisition software. Image analysis was done using the Image-Pro Plus 7.0 (Media Cybernetics, Rockville, MD) software.

### Differentiation assay

Differentiation assays were performed as previously described with the following change^34^. Cells were grown in complete neural basal media containing 1% fetal calf serum. Image acquisition was performed using the Leica DMI 4000B microscope (Leica Microsystems, Wetzlar, Germany) with Q Imaging Retiga-SRV Fast 1394 camera (Q Imaging, Surrey, Canada) and Surveyor 7.0.0.9 (Objective Imaging, Kansasville, WI) imaging acquisition software.

### Real-time PCR

RNA was extracted using RNeasy Mini Prep Kit (Qiagen, Redwood, CA) and cDNA made using the High Capacity cDNA Reverse Transcription Kit (Life Technologies, Grand Island, NY). qPCR was performed using the 7900HT Fast Real-Time PCR System (Life Technologies, Grand Island, NY) and the data analyzed with the ABI7900HT Sequence Detection Software v2.4. (Life Technologies, Grand Island, NY). All qPCR primer pairs were obtained from the RT^2^ qPCR Primer Assay system generated by Qiagen (Qiagen, Redwood, CA).

### Statistical Analysis

Survival curves were generated using the Kaplan-Meier method. The survival of mice in the different treatment groups was compared using the log-rank test. *P* < 0.05 was considered statistically significant. All statistical analysis was performed using Prism 6 for Mac OSX (v.6.0c) (GraphPad Software, La Jolla, CA) in Data Analysis using Regression or Student's t-Test: Paired Two Sample for Means. Probabilities for the Student's t-Test are listed as “P(T ≤ t) two-tail” with an alpha of 0.05.

### Vertebrate Approval

All experiments involving live vertebrates were in compliance with and preformed under the guidelines of a Nationwide Children's Hospital IACUC approved protocol.

## Author Contributions

R.J. wrote main manuscript text and contributed to all figures. P.C. and N.R. contributed to preparation of figures 1, 2 and 6. S.P. contributed to figures 4 and 5. A.S. and C.P. contributed to figure 3. All authors reviewed the manuscript.

## Supplementary Material

Supplementary Information Supplementary Data
