# Supplementary material for: EphB2 activation is required for ependymoma development as well as inhibits differentiation and promotes proliferation of the transformed cell
Source: Sci Rep. 2015 Mar 24;5:9248. doi: 10.1038/srep09248 (PMC4371088; doi:10.1038/srep09248)

Title: EphB2 activation is required for ependymoma development as well as inhibits differentiation and promotes proliferation of the transformed cell

Authors: Phylip Chen*, Nathan Rossi*, Samuel Priddy, Christopher R. Pierson[#], Adam W. Studebaker*, Robert A. Johnson*[¶]

Supplemental Figure 1: **(a)** Ephrin-B1 and ephrin-B2 western blot of uninfected Ink4a/Arf$^{(-/-)}$ STeNSCs (lane 3) or infected with control (pCX4-RedEX; lane 1) or EphB2 expressing (lane 2) retrovirus. **(b)** Ligand concentration titration experiment: *In vitro* EphB2 activation assay of utNSCs treated with anti-human-IgG (lane 1) or increasing concentration of ephrin-B1 (0.1, 0.25, 0.5, 1.0, 2.0μg/ml; lanes 2-6). **(c)** EphB2 receptor activation IP/western after ephrin-B(1-3) treatment for 24 or 48hrs.

Supplemental Figure 2: Chart of total luciferase activity (total flux (p/s)) over time (days) for mice intracranially implanted with Ink4a/Arf$^{(-/-)}$ STeNSCs co-infected with MSCV-Luc and either EphB2, EphB2(K662R) or EphB2(ΔSAM/PDZ). Values in red indicate the last measurement and day of sacrifice before experiment termination at 400 days.

Supplemental Figure 3: **(a)** Chart of the percentage of each neuronal cell type identified by both linage marker expression and morphology of Ink4a/Arf$^{(-/-)}$ STeNSCs infected with nothing, EphB2 (utNSCs) or EphB2(K662R). Graphs depicting the percent of Ink4a/Arf$^{(-/-)}$ STeNSCs **(b)** or EphB2(K662R) infected Ink4a/Arf$^{(-/-)}$ STeNSCs **(c)** differentiated into astrocyte (βIII-tubulin), neuron (S100β) or oligodendrocytes (NG2) after treatment with nothing (untreated), anti-human-IgG, ephrin-B2 or ephrin-B3. Neuronal type determined

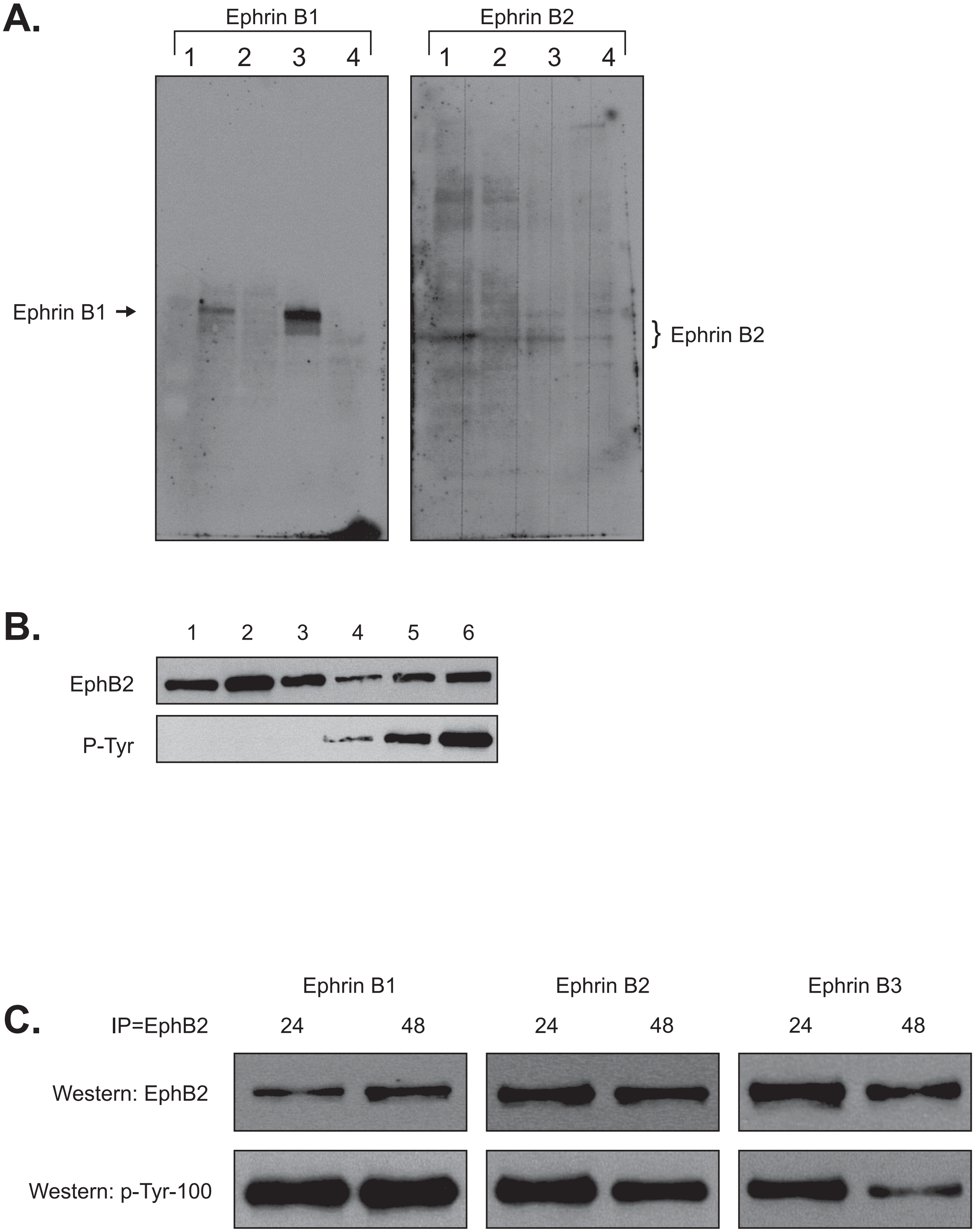
A.
Ephrin B1
1 2 3 4
Ephrin B2
1 2 3 4
Ephrin B1
Ephrin B2
B.
1 2 3 4 5 6
EphB2
P-Tyr
C.
Ephrin B1
Ephrin B2
Ephrin B3
IP=EphB2
24 48 24 48 24 48
Western: EphB2
Western: p-Tyr-100

Days	11	30	121	151	161	169	197	259	372
K66R-RA	1.18E+06	4.90E+05	8.18E+05	9.87E+05	1.42E+06	9.65E+05	7.82E+05	1.25E+06	2.68E+04
K66R-RM	4.00E+05	2.22E+05	2.82E+05	4.08E+05	2.71E+05	2.57E+05	2.04E+05	2.54E+05	2.31E+05
K66R-RP	3.35E+06	9.97E+05	3.87E+04	3.04E+04	3.25E+04	3.82E+04	3.17E+04	1.80E+04	4.67E+05
K66R-LA	2.30E+06	6.61E+05	7.08E+05	9.55E+05	1.53E+06	1.02E+06	7.73E+05	9.21E+05	1.45E+06
K66R-LP	4.09E+05	1.84E+05	4.94E+05	3.50E+05	5.18E+05	3.11E+05	4.66E+05	5.34E+05	1.68E+06

Days	11	30	121	151	161	169	185	197	210	234	259	284	298	301	340	354	372
dSAM/PDZ-RA	3.14E+05	2.90E+05	2.57E+06	3.98E+06	5.78E+06	5.43E+06	1.11E+05	4.51E+06	5.81E+06	7.75E+06	1.08E+07						
dSAM/PDZ-RM	4.11E+06	7.26E+06	3.11E+06	5.48E+06	4.20E+06	6.73E+06	9.37E+06	4.17E+06	2.20E+06	8.65E+06	1.34E+07	8.82E+06		1.51E+07	2.06E+07	2.55E+07	2.65E+07
dSAM/PDZ-RP	1.81E+06	1.41E+06	3.55E+06	5.03E+06	5.88E+06	9.22E+06	1.60E+07	1.07E+07	1.23E+07								
dSAM/PDZ-LA	9.31E+05	4.03E+05	4.59E+05	6.54E+05	1.36E+06	1.86E+06	3.33E+06	2.85E+06	1.97E+06	3.88E+06	5.74E+06	6.30E+06	4.18E+07				
dSAM/PDZ-LP	1.22E+06	8.71E+05	1.42E+06	2.34E+06	7.21E+06	6.60E+06	9.99E+06	1.28E+07	3.49E+07								

Days	13	30	78	105	137	145	159	173	187	211	250	274	298	337	363
EphB2-RA	1.44E+06	5.89E+06	9.44E+05	3.66E+07	5.83E+07	4.65E+07	6.54E+07	6.48E+07	9.16E+07	1.23E+08	4.71E+08				
EphB2-RM	1.28E+06	1.54E+06	4.39E+06	1.14E+08	1.59E+08	1.35E+08	2.46E+08	3.13E+08	6.01E+08						
EphB2-RP	1.10E+07	5.30E+07	8.52E+06	6.19E+07	1.06E+08	5.44E+07	8.93E+07	1.43E+08							
EphB2-LA	1.83E+06	1.01E+07	1.28E+06	1.70E+07											
EphB2-LP	1.89E+06	9.81E+06	1.55E+06	1.70E+07	2.32E+07	1.63E+07	3.66E+07	2.90E+07	2.90E+07	7.71E+07	1.50E+08	5.10E+08	9.41E+08	3.42E+09	4.86E+09

A.

utNSCs Differentiation Assay (%)

	Untreated	Human IgG-FC	ephrin-B2Fc	ephrin-B3-Fc
βIII-Tubulin	4.781	3.669	7.953	10.21
S100β	52.69	55.55	57.46	52.26
NG2	37.58	35.75	35.92	38.53

EphB2(K662R) Differentiation Assay (%)

	Untreated	Human IgG-FC	ephrin-B2Fc	ephrin-B3-Fc
βIII-Tubulin	8.045	5.62	7.946	11.09
S100β	59.06	55.18	49.29	58.59
NG2	36.91	41.67	31.32	43.33

Ink4a/Arf $^{(-/-)}$ Differentiation Assay (%)

	Untreated	Human IgG-FC	ephrin-B2Fc	ephrin-B3-Fc
βIII-Tubulin	12.75	14.59	17.92	14.22
S100β	50.27	56.26	62.62	57.48
NG2	40.45	45.87	41.98	45.95

B.

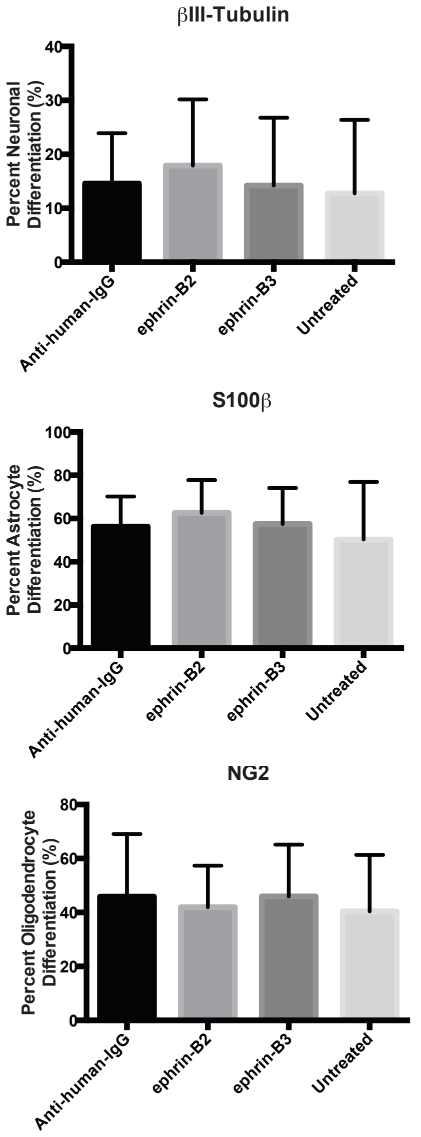

C.

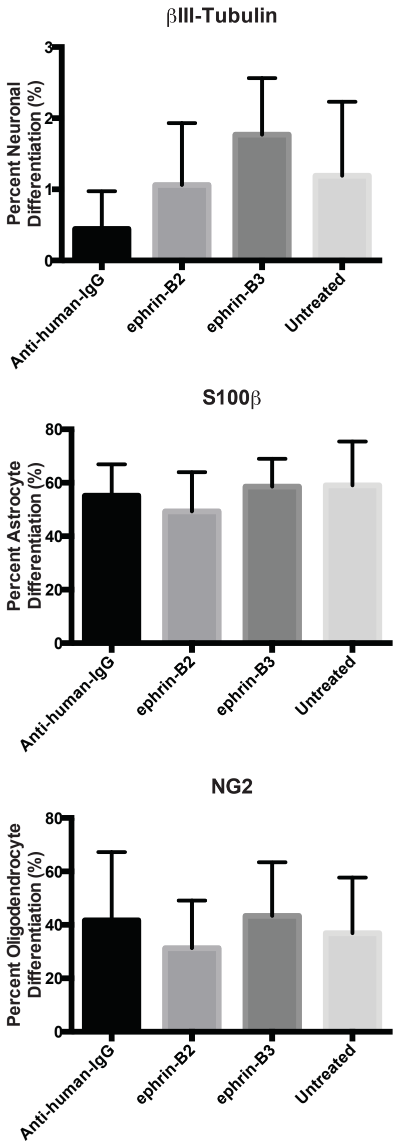

Supplement: Supplementary Information — Supplementary Data [file srep09248-s1.pdf]
